# Retraction: Overexpression of long intergenic noncoding RNA LINC00312 inhibits the invasion and migration of thyroid cancer cells by down-regulating microRNA-197-3p

**DOI:** 10.1042/BSR-2017-0109_RET

**Published:** 2021-06-08

**Keywords:** invasion and migration, Long intergenic noncoding RNA LINC00312, MicroRNA-197-3p, Thyroid cancer

This Retraction follows an Expression of Concern relating to this article previously published by Portland Press.

This article is being retracted from *Bioscience Reports* at the request of the Editor-in-Chief and the Editorial Board following receipt of a notification from a reader, alerting the Editorial Board to duplications in Figure 5, which also overlap with images published in *Journal of Cellular Biochemistry* (10.1002/jcb.26763) and in *Bioscience Reports* (10.1042/bsr20160542).

The authors have been contacted in regards to the retraction, and have not responded to the Journal's queries and the concerns raised. Given the extent of the issues raised, the Editorial Board stand by the decision to retract the article.

